# Feature reliability determines specificity and transfer of perceptual learning in orientation search

**DOI:** 10.1371/journal.pcbi.1005882

**Published:** 2017-12-14

**Authors:** Amit Yashar, Rachel N. Denison

**Affiliations:** 1 Department of Psychology and Center for Neural Science, New York University, New York, New York, United States of America; 2 The School of Psychological Sciences, Tel Aviv University, Tel-Aviv, Israel; University of Birmingham, UNITED KINGDOM

## Abstract

Training can modify the visual system to produce a substantial improvement on perceptual tasks and therefore has applications for treating visual deficits. Visual perceptual learning (VPL) is often specific to the trained feature, which gives insight into processes underlying brain plasticity, but limits VPL’s effectiveness in rehabilitation. Under what circumstances VPL transfers to untrained stimuli is poorly understood. Here we report a qualitatively new phenomenon: intrinsic variation in the representation of features determines the transfer of VPL. Orientations around cardinal are represented more reliably than orientations around oblique in V1, which has been linked to behavioral consequences such as visual search asymmetries. We studied VPL for visual search of near-cardinal or oblique targets among distractors of the other orientation while controlling for other display and task attributes, including task precision, task difficulty, and stimulus exposure. Learning was the same in all training conditions; however, transfer depended on the orientation of the target, with full transfer of learning from near-cardinal to oblique targets but not the reverse. To evaluate the idea that representational reliability was the key difference between the orientations in determining VPL transfer, we created a model that combined orientation-dependent reliability, improvement of reliability with learning, and an optimal search strategy. Modeling suggested that not only search asymmetries but also the asymmetric transfer of VPL depended on preexisting differences between the reliability of near-cardinal and oblique representations. Transfer asymmetries in model behavior also depended on having different learning rates for targets and distractors, such that greater learning for low-reliability distractors facilitated transfer. These findings suggest that training on sensory features with intrinsically low reliability may maximize the generalizability of learning in complex visual environments.

## Author summary

Training can modify the visual system to produce improvements on perceptual tasks (visual perceptual learning), which is associated with adult brain plasticity. Visual perceptual learning has important clinical applications: it improves the vision of adults with visual deficits, e.g. amblyopia and cortical blindness, and even presbyopia (aging eye). A critical issue in visual perceptual learning is its specificity to the trained stimulus. Specificity gives insight into the processes underling experience-dependent plasticity but can be an obstacle in the development of efficient rehabilitation protocols. Under what circumstances visual perceptual learning transfers to untrained stimuli is poorly understood. Here we report a qualitatively new phenomenon: specificity in visual search depends on intrinsic variations in the reliability of feature representations; e.g., vertically oriented lines are represented in V1 with greater reliability than tilted lines. Our data and computational model suggest that training on sensory features with intrinsically low reliability can maximize the generalizability of learning, particularly in complex natural environments in which task performance is limited by low-reliability features. Our study has possible implications for the development of efficient clinical applications of perceptual learning.

## Introduction

Training in fundamental visual perceptual tasks can lead to substantial improvement, a phenomenon known as Visual Perceptual Learning (VPL), which is associated with adult brain plasticity. VPL has powerful real-word applications [1–3] including improving the vision of adults with cortical blindness [4], amblyopia [5–7] and presbyopia [8].

VPL is often specific to the trained feature and location (reviewed by [9]). From a theoretical point of view, specificity can provide important insight into the neuronal mechanisms that underlie VPL. For example, specificity has been taken to imply plasticity in early-stage visual processing (e.g., [10,11]). However, from a practical or clinical viewpoint, specificity can be a major obstacle in the development of effective training protocols, and it is therefore critical to understand the factors that determine VPL specificity and the conditions that lead to transfer.

For complete transfer to occur, the visual system needs to apply learning for one stimulus to another stimulus. The ability to generalize improvements across stimuli may be most likely when the representation of the stimuli is intrinsically similar. However, the visual system has intrinsic variations in its representation of different feature values. In particular, the reliability with which different feature values are represented can vary considerably within a feature dimension. For example, the reliability of orientation representation in V1 strongly depends on the orientation value. Cells responding to orientations around cardinal are larger in number and have smaller response variability compared to cells responding to orientations around oblique [12,13]. In human V1-V3, sensory uncertainty estimated from the fMRI BOLD signal is higher near oblique orientations than near cardinal orientations, which correlates with orientation estimation behavior [14]. These studies show a gradual variation in representational reliability as a function of orientation, with higher reliability for orientations closer to cardinal (especially horizontal) and lower reliability for more oblique orientations.

These intrinsic differences have been linked to substantial behavioral effects unrelated to learning. They explain the advantage that observers have in discriminating orientations around cardinal compared to around oblique [13–16]: the oblique effect [17]; and in detecting oblique targets among cardinal [18–20] or near-cardinal [21] distractors over the reverse: orientation search asymmetry [22]. Explanations of search asymmetry propose that oblique distractors have less reliable representations than cardinal distractors and thus hinder target detection more [19,20]. Intrinsic variations in representational reliability are not limited to orientation; for example, stimulus processing also varies across spatial frequency [23]. Thus far, however, no study has directly investigated the effect of these preexisting variations in representational reliability on VPL transfer and specificity.

Investigation of VPL has focused instead on the manipulation of task properties. By varying task difficulty [10,24] and task precision (e.g., orientation difference in a discrimination task; [25]), researchers varied the representational precision required to successfully perform the task, and studied its effect on VPL specificity. However, variability in task demands is distinct from initial variability in the underlying representation and may invoke different learning mechanisms. For example, increased specificity in difficult or high-precision tasks has been attributed to changes in the modulated level of representation in the visual processing hierarchy [10,24], whereas intrinsic differences in representational reliability are present within the same hierarchical level.

Here, we asked whether variations in representational reliability alone can explain VPL and its specificity and transfer, when task properties such as difficulty and precision are the same. Our results show that near-cardinal and oblique orientations not only yield an orientation search asymmetry [18–22] but also show asymmetric transfer of VPL in visual search. Conversely, task difficulty, which was independently manipulated by varying the stimulus onset asynchrony (SOA) between a mask and the search display, did not affect the pattern of transfer. To test the sufficiency of a reliability-based account, we fit a computational model that combines learning-related increases in the reliability of stimulus representations with a Bayesian search strategy based on Ma et al. [26]. This Bayesian search model was well-suited to test our hypothesis, because it explicitly represents orientation reliability. Using an unchanging optimal decision rule, the model accounts for both search and transfer asymmetry via initial differences in near-cardinal and oblique orientation reliability.

## Results

**Fig 1:**
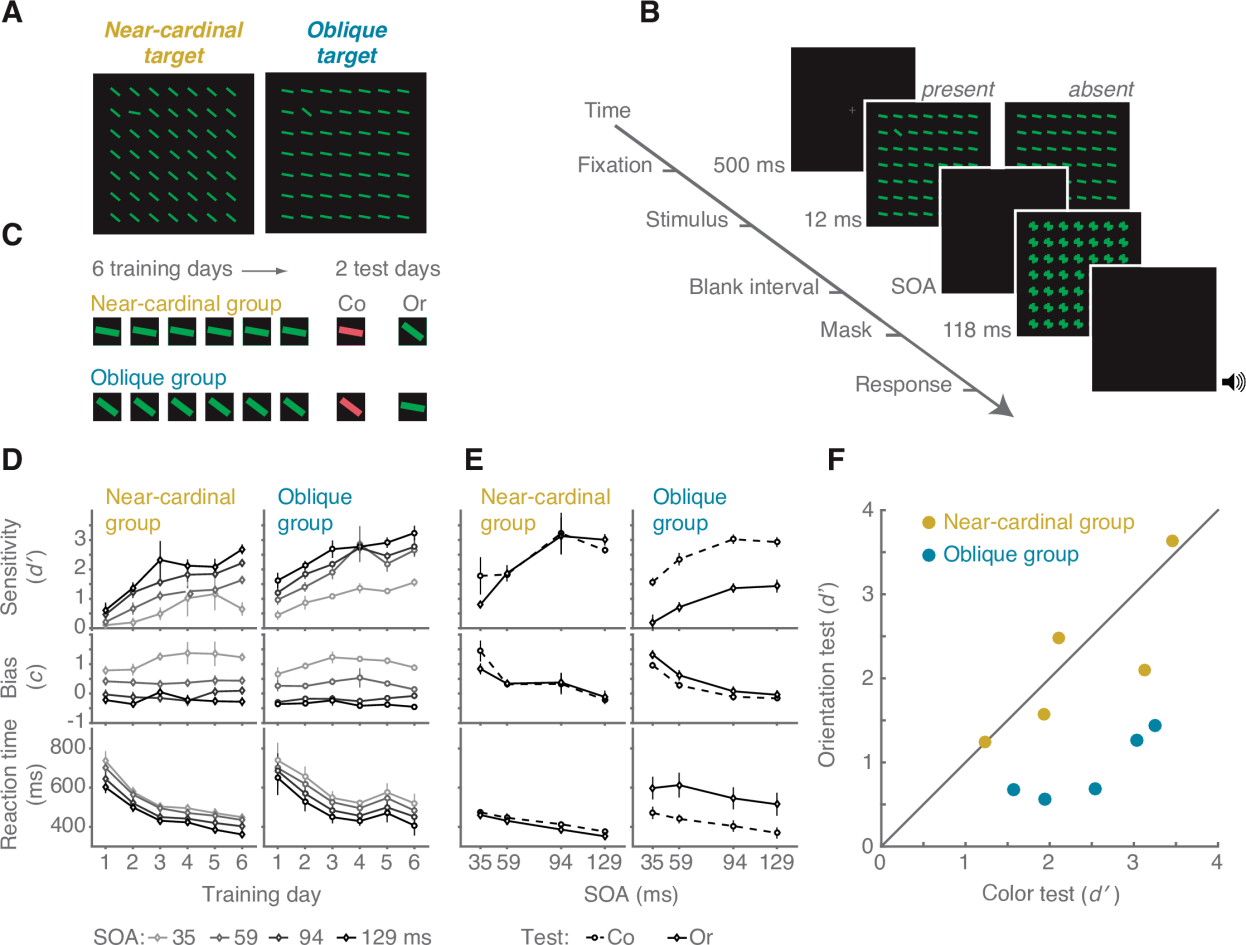
Experimental design and results. (A) The search stimulus in the near-cardinal target, 80° (left) and the oblique target, 50° (right) conditions. Stimulus color was either red or green on training days and for the orientation test (green in the example shown) and the other color for the color test. (B) The sequence of events within a trial. (C) Training protocol: the near-cardinal group was trained with a near-cardinal target and tested with an oblique target and vice versa for the oblique group. The order of the color and orientation test days was counterbalanced. (D) Mean sensitivity, bias and RT (y-axis top, middle and bottom panels respectively) as a function of training day (x-axis) and SOA, for both near-cardinal (left) and oblique (right) groups. Performance increased with training. (E) Mean sensitivity, bias and RT as a function of SOA (x-axis) and color and orientation transfer tests, for both near-cardinal (left) and oblique (right) groups. Performance was lower in the orientation test compared to the color test only in the oblique group. Error bars are within-subject ±1 standard error of the mean [27]. (F) Scatterplot for individual observers (mean across SOAs), d′ for orientation training performance during the color transfer test (color test) and orientation transfer performance (orientation test) for the near-cardinal and oblique groups. Data points near the unity line indicate transfer, while data points below the unity line indicate specificity.

We trained observers in visual search for an odd orientation (Fig 1A & 1B). One group of observers trained with a near-cardinal target and oblique distractors (near-cardinal group) and the other group trained with an oblique target and near-cardinal distractors (oblique group). Near-cardinal was 80° counter-clockwise from vertical and oblique was 50° from vertical. In both groups, the search stimulus color (task irrelevant) was either green or red. Following training, observers completed an orientation test and a control, color test. In the orientation test, only the orientation swapped with respect to training, i.e. the near-cardinal group was tested with the oblique target and the oblique group was tested with the near-cardinal target (Fig 1C). In the color test, only the color of the stimulus was different with respect to training, i.e. observers that trained with red stimuli were tested with green stimuli and vice versa. Comparing these tests controlled for the involvement of high-level cognitive factors during test sessions (e.g. observers are more engaged due to any new aspect of the stimuli). Perceptual sensitivity (d′) and bias (c) were calculated for each SOA in each session. Incorrect trials and trials with reaction time (RT) ≥4 SDs above the observer’s mean (≤0.5% of the trials) were removed from the RT analysis.

### Training sessions

To test for learning, we compared the first and the last training days. For all three dependent measurements (sensitivity, bias and RT) we conducted a (2X2X2) three-way mixed design analysis of variance (ANOVA) with training effect (training day 1 vs. 6) and SOA (35, 59, 94 and 129 ms) as within-observers factors and group (near-cardinal vs. oblique training) as a between-observers factor.

#### Perceptual sensitivity

The main effect of training was significant, F(1, 8) = 93.30, p<0.001, $\eta_{p}^{2}$ = 0.92, with higher perceptual sensitivity on the last compared to the first training day (Fig 1D), indicating learning. The main effect of SOA was significant, F(3, 24) = 39.73, p<0.001, $\eta_{p}^{2}$ = 0.43, with higher sensitivity for longer SOAs. The main effect of group was significant, F(1, 8) = 5.41, p = 0.048, $\eta_{p}^{2}$ = 0.47, with higher sensitivity in the oblique group than in the near-cardinal group (orientation search asymmetry). Only the interaction between training and SOA was significant, F(3, 24) = 7.21, p = 0.001, $\eta_{p}^{2}$ = 0.40, indicating more benefit from training for longer compared to shorter SOAs. None of the effects interacted with group, all ps>0.1, indicating that the learning and SOA effects were similar in both groups. To confirm that learning was the same across groups, we estimated the learning rate for each observer as the slope of a linear regression for d’ across the six training days. Learning rates were the same for the near-cardinal group (mean slope: 0.28 ±0.03) and the oblique group (mean slope: 0.28 ±0.04), t<1.

#### Bias

The main effect of SOA was significant, F(3, 24) = 33.30, p<0.001, $\eta_{p}^{2}$ = 0.80, with more conservative bias with shorter SOAs. Neither the interaction between SOA and training, p>0.05, nor the remaining comparisons, ps>0.3, were significant.

#### RT

Training speeded RT, F(1, 8) = 19.74, p = 0.002, $\eta_{p}^{2}$ = 0.71, as did longer SOAs, F(3, 24) = 19.61, p<0.001, $\eta_{p}^{2}$ = 0.71 (Fig 1D). None of the other effects were significant for RT, all ps>0.05. These results confirm that the effects on perceptual sensitivity were not due to tradeoffs with RT.

### Testing sessions

To test for the transfer of learning for each of the three dependent measurements, we conducted a (2X2X2) three-way mixed design ANOVA with tests (color test vs. orientation test) and SOA (35, 59, 94 and 129 ms) as within-observers factors and group (near-cardinal vs. oblique group) as a between-observers factor. As Fig 1E reveals, whereas color test performance was very similar to the last day of learning in both groups, orientation test performance was dependent on the group.

#### Perceptual sensitivity

**Fig 2:**
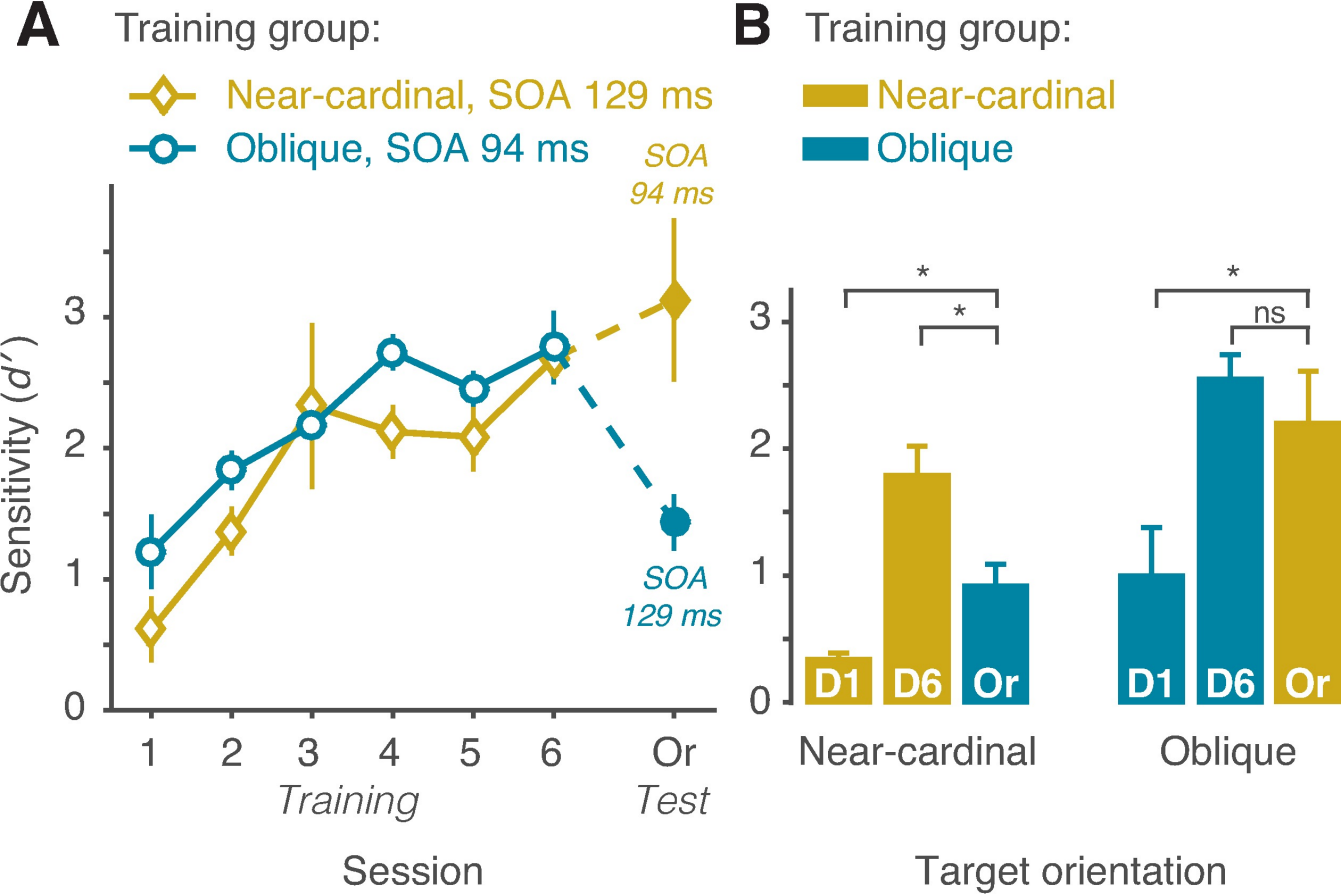
Orientation specificity and transfer: between-group comparisons. (A) Sensitivity for each group as a function of training day and orientation test. Training performance was equated by choosing SOA of 129 ms and 94 ms for the near-cardinal and oblique groups, respectively. (B) Sensitivity averaged across SOAs as a function of session (D1: first training day, D6: last training day, Or: orientation test) and target orientation (near-cardinal and oblique). Certain comparisons are across groups (yellow vs. blue bars). ns = p>0.4, * = p<0.05. Error bars are within-subject ±1 standard error of the mean [27].

The main effect of test was significant, F(1, 8) = 31.45, p = 0.004, $\eta_{p}^{2}$ = 0.79, with higher sensitivity in the color test than in the orientation test. Again, the main effect of SOA was significant, F(3, 24) = 27.56, p<0.001, $\eta_{p}^{2}$ = 0.90, with higher perceptual sensitivity for longer SOAs. The main effect of group was not significant, F(1, 8) = 1.6, p>0.2. Importantly, the interaction between test and group was significant, F(1, 8) = 20.30, p = 0.002, $\eta_{p}^{2}$ = 0.70, indicating that transfer across tests was dependent on training target orientation. Paired t-tests revealed that sensitivity in the orientation test was lower than in the color test only in the oblique group, t(4) = 8.53, p = 0.001, Cohen’s d = 2.6, and not in the near-cardinal group, t<1. None of the other interactions was significant, all ps>0.3. Individual data show consistent specificity of improvement in the oblique group and transfer in the near-cardinal group (Fig 1F). In both groups, VPL completely transferred to a new color (Fig 1D & 1E), confirming that observers learned orientations independent of their colors. Importantly, the group effect on orientation transfer remained the same even when task difficulty was equated by selecting an SOA for each group that yielded similar performance (d′) for the two groups during training (Fig 2A).

#### Bias

The main effect of SOA was significant, F(3, 24) = 20.31, p<0.001, $\eta_{p}^{2}$ = 0.71, with more conservative bias with shorter SOAs. Although both test and group effects were not significant F<1, the interaction between the two factors approached significance levels, F(1, 8) = 5.19, p = 0.052, $\eta_{p}^{2}$ = 0.39. Paired comparisons between the orientation test and the color test for each group revealed a test effect that approached significance levels in the oblique group, t(4) = 2.55, p = 0.063, with more conservative bias in the orientation test than in the color test, M = 0.49 and 0.24, respectively. No such effect was found in the near-cardinal group, t<1.

#### RT

RTs were faster in the color than in the orientation test, F(1, 8) = 48.24, p<0.001, $\eta_{p}^{2}$ = 0.87, and for longer SOAs, F(3, 24) = 33.96, p<0.001, $\eta_{p}^{2}$ = 0.81. The main effect of group was not significant, F(1, 8) = 1.6, p>0.2. The interaction between test and group was significant, F(1, 8) = 92.51, p < .001, $\eta_{p}^{2}$ = 0.93. Paired t-tests between color and orientation tests revealed that in the oblique group RTs were slower in the orientation than in the color test, t(4) = 8.56, p = 0.001, Cohen’s d = 1.1. Conversely, in the near-cardinal group RTs were slightly slower in the color than in the orientation test, t(4) = 3.48, p = 0.02, Cohen’s d = 0.2.

### Transfer and specificity

Because baseline performance (training day 1) for the near-cardinal condition was lower than for the oblique condition, it may be that during the orientation test (when target and distractor orientations swapped) specificity was inflated by the baseline difference. In order to control for this possibility, we additionally assessed transfer and specificity by comparing performance (d′) in the orientation transfer test from one group with the baseline performance (training day 1) and trained performance (training day 6) of the other group, such that the orientation condition was the same within each comparison (Fig 2B). First we tested whether transfer performance is higher than baseline, which would indicate that at least some learning partially transferred to the untrained orientation. Two independent sample one-tailed t-tests revealed significant transfer both to near-cardinal and to oblique orientations, t(8) = 3.08, p = 0.007, Cohen’s d = 1.94, t(8) = 2.01, p = 0.039, Cohen’s d = 1.28, respectively. Next we tested whether transfer performance is different than trained performance; a difference would indicate specificity, while no difference would indicate full transfer of learning to the untrained orientation. Two independent sample t-tests revealed significant partial specificity following oblique orientation training, t(8) = 2.96, p = 0.018, Cohen’s d = 2.05, but not following near-cardinal orientation training, t<1. The same results were obtained when a nonparametric test was used (S1 Table). Thus, learning only partly transferred to the near-cardinal orientation but fully transferred to the oblique orientation.

### Model

Because we found that VPL specificity and transfer depended on the trained orientation–despite equated task difficulty and task precision–we hypothesized that differences in the representational reliability of near-cardinal and oblique orientations may lead to both search and VPL transfer asymmetries. To investigate this possibility, we used computational modeling. We developed a model that consists of two parts: optimal orientation search [26,28] and reliability improvement over the course of learning. The goal was to determine whether orientation reliability and its improvement with learning could explain the behavioral data.

We compared four models to test different hypotheses about the role of orientation reliability in learning and transfer in the orientation search task. We tested whether initial reliability differences between near-cardinal and oblique orientations alone (Reliability model), different learning rates for targets and distractors alone (Learning model), both of these factors together (Reliability-and-Learning model), or these factors with independent learning rates for the two groups (Reliability-Learning-Group model) best accounted for the data. Detailed descriptions of the models can be found in the Methods, and all model fits are shown in S1 Fig.

Model comparison using the AICc metric indicated that initial reliability differences between near-cardinal and oblique orientations were critical to explain the data. The Reliability model (three parameters, AICc = 10.61) and the Reliability-and-Learning model (four parameters, AICc = 13.00) outperformed the Learning model (three parameters, AICc = 21.05) and the Reliability-Learning-Group model (six parameters, AICc = 24.56). When we compared cross-validated r^2^, the Reliability-and-Learning model fit the data better than the Reliability model. For the Reliability-and-Learning model, cross-validated r^2^ was 0.81 (SD 0.09), falling within the noise ceiling (lower and upper bounds, [0.75 0.84]), Model performance was therefore as good as possible given the noise in the data. For the Reliability model, cross-validated r^2^ was 0.70 (SD 0.24), falling below the noise ceiling.

**Fig 3:**
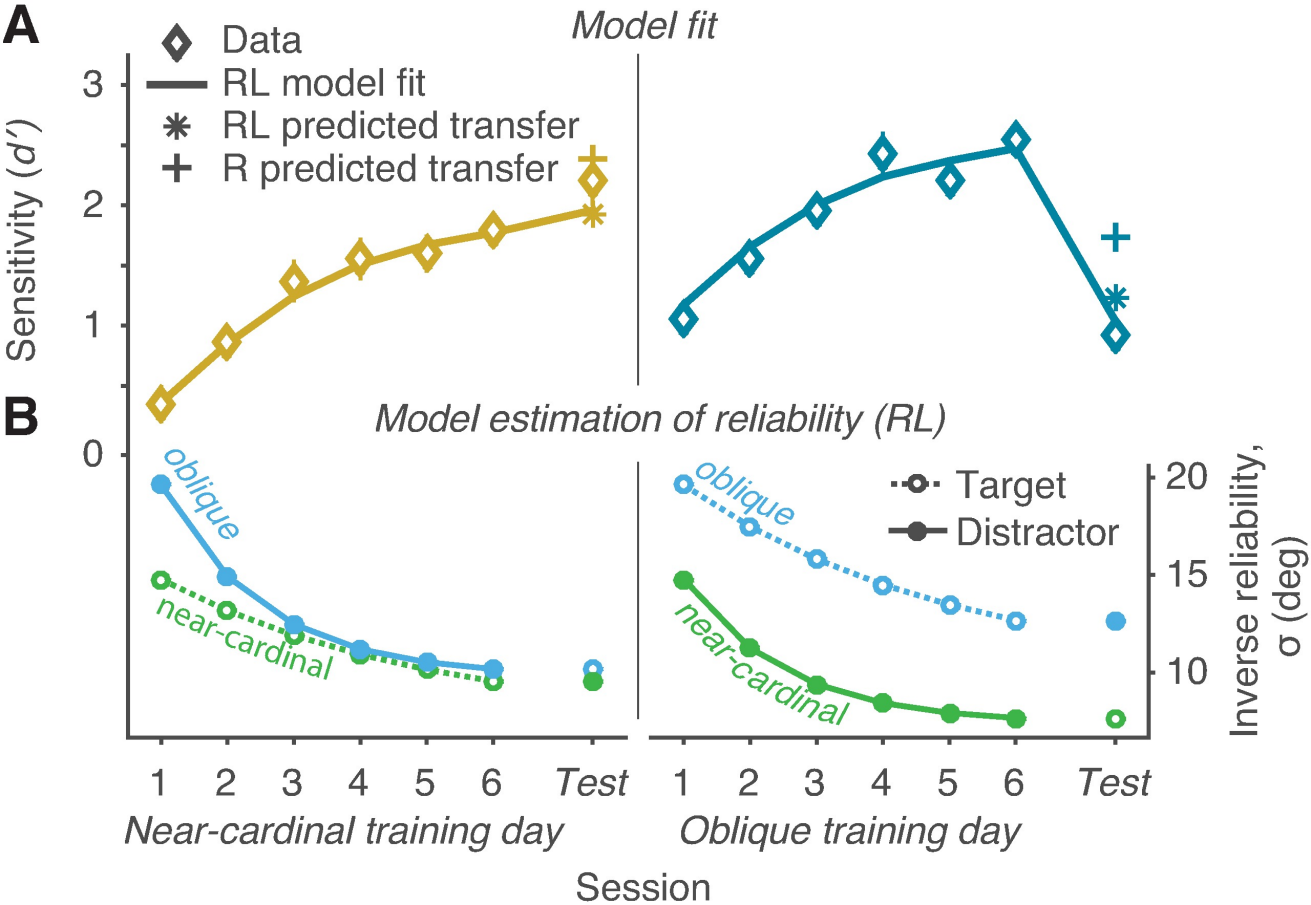
Model performance and estimated reliability for the near-cardinal (left column) and oblique (right column) groups. (A) Curves show the Reliability-and-Learning model fit to the sensitivity data (diamonds) averaged across SOAs for each training day (1–6) and orientation transfer test (Or). Reliability-and-Learning (stars) and Reliability (plus signs) show transfer test predictions based on fits to the training days only. Error bars are within subject ±1 standard error of the mean [27]. (B) Estimated inverse reliability (σ) for the target and distractor stimulus representations as a function of training day and orientation test. RL: Reliability-and-Learning model; R: Reliability model.

To determine whether transfer and specificity in the two best models could be predicted based only on the learning phase, we fit the models to the training days only and predicted the transfer test performance for each group. For the Reliability-and-Learning model, the predicted orientation test performance was similar to the observed performance, namely, transfer in the near-cardinal group and specificity in the oblique group (Fig 3A, stars). The Reliability model predicted more transfer in the oblique group than was observed in the data (Fig 3A, plus signs), similar to its fit to all data points (S1 Fig). The pattern of transfer and specificity therefore did not depend on including the test session data when fitting the model, and the Reliability-and-Learning model better explained transfer behavior.

The Reliability-and-Learning model, then, captured the three key features of the data: 1) the search asymmetry at baseline, 2) the performance improvement with learning, and 3) the orientation dependence of VPL specificity and transfer (Fig 3A). Learning in the oblique group maintained the difference in reliability between the near-cardinal and oblique orientations, thereby maintaining the search asymmetry present at baseline and preventing full transfer. Conversely, learning in the near-cardinal group decreased the reliability difference between orientations, effectively overcoming the search asymmetry and allowing similar near-cardinal and oblique performance by the end of training.

Fig 3B shows Reliability-and-Learning model estimates of near-cardinal and oblique reliability as a function of training session for each group. The model estimated greater sensory uncertainty (lower reliability) for the oblique than for the near-cardinal orientation, consistent with physiological and behavioral findings [12,13]. For the best-fitting parameter estimates, the distractor learning rate was 0.65 and the target learning rate was 0.24.

## Discussion

### Learning and specificity

Existing models of VPL predict the same level of specificity across the same levels of task-difficulty [24], task precision [25,29] and feature exposure during training [30]. The demonstration that a mere difference in the trained feature value, near-cardinal vs. oblique orientation, determined VPL specificity challenges these views. Supported by computational modeling, we suggest that intrinsic differences in the representational reliabilities of near-cardinal and oblique orientations governed VPL specificity and transfer in orientation search.

Our design enabled us to control for the involvement of task-related factors and to assess the effect of representational reliability per se. In both groups the equal orientation difference between targets and distractors (30°), equated performance controlled by SOA, and identical exposure to the transfer feature insured independence from task precision [25], difficulty [24], and feature exposure [30], respectively. Our analyses confirmed that both learning rate and magnitude were equal for the two groups. In addition, our results cannot be explained in terms of differences in number of difficult trials during training. A larger number of difficult trials during training has been found to increase specificity [31]. This relationship would predict a result opposite to ours: specificity in the near-cardinal group, which was more difficult on average (across SOAs). Thus, stimulus-related properties, rather than task, determined specificity here.

The dependence of transfer on the specific orientation value has implications for the investigation and interpretation of VPL transfer and specificity using oriented stimuli. Indeed such stimuli have been commonly used to investigate VPL, including in orientation discrimination tasks (e.g., [25,30,32–34]), visual search (e.g., [10,30,35,36]) and texture discrimination tasks (e.g., [37–41]). Some VPL studies have varied orientation values to manipulate task properties, such as task difficulty, and then linked those task properties to the resulting feature specificity (e.g., [10,30,37]). Our study suggests that orientation differences alone can affect the pattern of feature specificity and transfer and therefore should be controlled, particularly in displays with more than one orientation.

Researchers have inferred the site of the underlying plasticity in VPL based on specificity and transfer results (reviewed by [42]). Specificity and transfer have been taken to indicate learning in early and late visual areas, respectively [10,11,24,30]. Here we show that preexisting variation in representational reliability, which can occur within the same level of processing, can determine VPL transfer. Our findings, therefore, suggest that specificity and transfer are not always appropriate diagnostic tools for the level of VPL plasticity.

### Implications of the model

Our model combined orientation-dependent reliability, improvement of reliability with learning, and an optimal search strategy. We based the search strategy on the optimal visual search model by Ma et al. [26], because that model provides a parsimonious explanation of orientation search with minimal parameters. We found that a single change to the model–letting reliability depend on orientation–captured orientation search asymmetry prior to learning. According to the model, the lower reliability of oblique compared to near-cardinal stimuli leads to more uncertainty during the local decision regarding the identity of an item. The disrupting effect of this uncertainty on visual search performance is larger with oblique distractors (near-cardinal target) than with near-cardinal distractors (oblique target), simply because there are many distractors but only one possible target in any given display.

The improvement of reliability across training days captured the behavioral pattern of both learning and transfer. Importantly, the model uses the same optimal decision rule throughout training and during the transfer tests. Search asymmetry and learning, therefore, could be attributed to variation in sensory reliability only, rather than changes in decision strategy and rule based learning [30].

Comparing alternative versions of the model allowed us to determine which factors were critical to explain the behavioral data. Preexisting differences in reliability were essential–a model without this component failed to fit the data–but independent learning for targets and distractors also improved model performance, particularly in capturing transfer behavior. This result is consistent with a previous study that found independent target and distractor learning in an orientation search task [43,44]. Our learning rate estimates correspond well to that study’s finding of about twice as much learning for distractors as for targets [43]. It is therefore the combination of preexisting reliability differences and greater learning for distractors than targets that best explained behavior, in this family of models. Specifically, greater learning for the initially low-reliability oblique distractors eliminated the search asymmetry and enabled full transfer for the near-cardinal group.

### Accounts of orientation search asymmetry

Our model follows the account that differences between the reliabilities of the cardinal (or near-cardinal) and oblique representations cause orientation search asymmetry [18–20]. A key component of these accounts is the ratio of target signal to background noise, which depends on the target and distractor identities [18,19,45]. Alternative accounts have also been proposed. One influential theory explains visual search asymmetries by considering a map of feature dimensions and their interactions [46]. This theory suggests that targets with larger feature values (e.g. more oriented, i.e. oblique) are inherently more detectable than targets with smaller values (e.g. less oriented, i.e. cardinal) (e.g., [46,47]). Based on this theory, a neural computational model was developed that explains search asymmetry in terms of a salience map in V1 [48]. However, it is unclear how the elimination of search asymmetry following near-cardinal training could be predicted if search asymmetry arises from inherent feature properties like “more tilted” [46,48,49]. Moreover, no previous model addresses VPL in orientation search.

### Feature reliability and its improvement in neural systems

Analogous to the reliability differences between orientation values represented by our model, neurons responding to oblique orientations have larger tuning curve widths than those responding to near-horizontal orientations in macaque V1 [13], and there is more cortical area tuned to near-cardinal orientations than to oblique orientations in ferret cortex [12]. Higher sensory uncertainty has also been estimated for oblique compared to near-cardinal orientations in human V1-V3 [14]. The Ma et al. [26] model on which the orientation search component of our model is based has been implemented as a biologically plausible neural network model, strengthening the connection between the physiological literature and our current computational results.

Learning was modeled as an increase in the representational reliability of the stimulus orientations. This increase could be implemented either as a reduction of the tuning curve width of V1 or V4 neurons with training [50–53] or as an improvement in readout from the early sensory response [29]. Both mechanisms have been proposed previously for an orientation discrimination task. Our model, therefore, applies VPL principles derived from orientation discrimination tasks to explain VPL for more complex visual search tasks.

### Limitations

Our findings are limited to VPL in orientation search, and more study is required to determine whether they generalize to other stimuli and tasks. Our study also does not rule out alternative models for orientation search asymmetry and VPL in visual search, but it shows that a parsimonious optimal decision rule, preexisting differences in orientation reliability, and reliability learning suffice to explain both search and transfer asymmetry.

For simplicity our model assumes the same representational reliability for all stimulus locations. However, stimulus reliability can vary as function of eccentricity (e.g., [18,23]) and polar angle [54,55]. It will be interesting to test the relation between location-dependent feature reliability and VPL transfer and specificity.

### Conclusions

Researchers have sought to understand the perceptual and neuronal processes that underlie VPL by studying how task demands affect VPL specificity. In the present study we control for task while testing the effect of the intrinsic reliability of feature representations on VPL specificity in visual search. We found a striking difference in VPL transfer depending on the orientation of the trained target, which we interpret as an effect of representational reliability. This interpretation is supported by both previous neurophysiological findings and computational modeling of the present data. We conclude that preexisting variation in the reliability of feature representations within a single level of processing may have a critical effect on VPL transfer and specificity, calling into question the logic that the degree of feature specificity can be used to infer the neural level at which VPL occurs, especially for complex visual displays.

A growing body of research demonstrates the potential benefits of VPL in clinical (e.g., [4–8,56,57]) and professional (e.g., [58]) applications. Our study suggests a testable hypothesis: to increase the generalizability of perceptual learning in real-world applications, efficient training protocols should focus training on low-reliability features–oblique orientations and motion directions [59], peripheral spatial locations [60], and so forth–which may limit performance in a variety of natural tasks.

## Methods

### Experimental procedures

#### Ethics statement

The Tel Aviv University Institutional Review Board approved this study.

#### Observers

Ten Tel-Aviv University students participated in the experiment (8 females, age range: 19–28 years) for course credit. All observers were naïve to the purposes of the study. All reported having normal or corrected-to-normal visual acuity and normal color vision.

#### Apparatus

Observers were tested individually in a dimly lit room. An Intel Core 2 Duo computer connected to a 17” CRT monitor (LG, with resolution of 768x1024 and refresh rate of 85 Hz). Stimuli were programmed in E-prime [61]. Responses were collected via the computer keyboard. A chin-rest was used to set viewing distance at 50 cm from the monitor.

#### Stimuli

Sample stimulus displays are presented in Fig 1A & 1B. The fixation display was a gray 0.38 x 0.38 degree of visual angle (dva) cross sign (+), in the center of a black background. The stimulus consisted of 49 tilted line-elements, drawn with 2 pixel stroke and subtending 0.58 dva in length. The lines were arranged in a 7X7 matrix centered on a black background. Each cell subtended 1.5 dva in side and each line was centered in its cell with a random jitter of ±0.01 dva. On target present trials (half of the trials) one line (the target) had a unique orientation and the remaining 48 lines (distractors) had a uniform orientation. On target absent trials (half of the trials) all 49 lines had the same orientation. Target position was randomly selected out of the 5X5 inner matrix cells (i.e. the target could not appear in the most peripheral rows and columns of the matrix). Target orientation always differed by 30° from that of distractors. There were two target conditions. Following Ahissar and Hochstein [62], in the near-cardinal target condition target and distractor orientations were 80° and 50° counterclockwise from vertical, respectively. In the oblique target condition target and distractor orientations were 50° and 80° counterclockwise from vertical, respectively (Fig 1A). Note that exact cardinal orientations (e.g., 0°) are at ceiling performance and do not improve with training in basic orientation discrimination tasks [63–65]. To avoid possible ceiling effects we used orientation values that differed from exact cardinal and oblique values by 10° and 5°, respectively. Such values still maintain a substantial variation in the representational reliability in V1-V3 [12–14] and as can be estimated from psychophysical measurements [21,65]. All lines were uniformly colored, ether red (CIE coordinates 0.63/0.34, 18.75 cd/m^2^) or green (CIE coordinates 0.28/0.593, 18.44 cd/m^2^). The mask consisted of 49 asterisk-like elements: a superposition of four line-elements each with different orientation (0°, 50°, 80°, 90°, counterclockwise). The positions and the color of the mask elements were the same as that of the stimulus lines.

#### Procedure

Each trial began with a 500 ms fixation display followed by the presentation of the stimulus for 12 ms, after which the screen went blank (Fig 1B). The mask stimulus was then presented for 118 ms. Stimulus onset asynchrony (SOA) between the search stimulus and the mask stimulus was 35 ms, 59 ms, 94 ms or 129 ms, a manipulation of task difficulty that would allow us to match difficulty for the two orientation conditions. All SOAs had the same number of trials, which were randomly interleaved within a session. Following the mask, the screen went blank until the observer made a response, with a maximum response window of 5000 ms. The task was a present/absent detection task. Observers were asked to report the presence of the target by pressing one of two designated keyboard keys (‘3’ for present and ‘z’ for absent target). Error trials were followed by a 500-ms feedback sound. Observers were instructed to respond as accurately as possible without speed stress. There was a 500-ms inter-trial interval. Observers were instructed to maintain fixation throughout each trial, and they had no time to make eye movements while the display was presented.

#### Design

There were eight experimental sessions of ~30-min each. Each session was held on a different day over the course of two weeks (4 sessions per week). Each session consisted of 540 trials. The first six days were training sessions and the last two days were test sessions (color test and orientation test). There were two groups of five observers each: One group trained with the near-cardinal target (near-cardinal group) and the other group trained with the oblique target (oblique group). In the color test, only the color (task irrelevant) of the stimulus was different with respect to training, i.e. observers that trained with red stimuli were tested with green stimuli and vice versa. In the orientation test, only the orientation swapped with respect to training, i.e. the near-cardinal group was tested with the oblique target and the oblique group was tested with the near-cardinal target (Fig 1C). The color of the training stimuli and the order of the test sessions were counterbalanced across observers. To reacquaint observers with the task, each session began with nine practice trials starting with long display duration (600 ms, 20 ms, and 12 ms for the first, middle, and last three trials of practice). During practice trials stimulus and mask were colored gray and target and distractor orientations were 160° and 100° counterclockwise respectively. Throughout the experiment observers were allowed a short break every 30 trials.

### Model

We developed a model that consisted of two parts: optimal search (based on Ma et al. [26]) and reliability improvement over the course of learning. We compared alternative versions of the model to determine which parameters were required to explain, in a single fit, the data from both observer groups, including the initial search asymmetry, performance improvement over the course of training, and the transfer asymmetry.

#### Optimal search

We adopted an optimal model of visual search developed by Ma et al. [26] that explains human behavior in an orientation search task similar to ours. The decision variable used to determine whether the target is present (T = 1) or absent (T = 0) on each trial is given by the log likelihood ratio

$$d=\log\frac{p(x_{1},\ldots ,x_{N}|T=1)}{p(x_{1},\ldots ,x_{N}|T=0)}$$ (1)

where x_i_ is the maximum-likelihood estimate of the stimulus orientation at location i, which we also call the orientation measurement. The total number of stimuli in the display is N, which was equal to 25 in our experiment and simulations (the inner 5x5 array where the target could appear). Because the target has a 0.5 probability of being present, the optimal strategy is to report that the target is present whenever d>0.

Each location is equally likely to contain the target. The decision variable for each trial can therefore be expressed as a combination of local decision variables, d_i_, for each stimulus location, as described by Ma et al. [26]:

$$d=\log\frac{1}{N}\sum_{i=1}^{N}e^{d_{i}}$$ (2)

Each local decision variable is the log likelihood ratio of the probability that the stimulus in that location is a target (s_i_ = s_T_) compared to the probability that it is a distractor (s_i_ = s_D_):

$$d_{i}=\log\frac{p(x_{i}|s_{i}=s_{T})}{p(x_{i}|s_{i}=s_{D})}$$ (3)

The value of s is 80° for near-cardinal orientations and 50° for oblique orientations.

The probability that the orientation measurement x_i_ for a given stimulus comes from a target or distractor depends on the orientation uncertainty (inverse reliability) of that stimulus, σ_i_. In the model of Ma et al. [26], σ_i_ depended only on stimulus contrast. Here, to model search asymmetries, we let σ_i_ depend on the orientation of the stimulus [20]. Near-cardinal orientations are expected to have lower uncertainty than oblique orientations [12,13]. We define σ_T_ as the orientation uncertainty of target stimuli and σ_D_ as the orientation uncertainty of distractor stimuli.

We assume that each of these probability distributions is Gaussian:

$$x_{i(t\arg et)}=N(s_{T},\sigma_{T}^{2})$$

$$x_{i(distractor)}=N(s_{D},\sigma_{D}^{2})$$ (4)

As derived in the Supplementary Methods in S1 Text, the local decision variable d_i_ is

$$d_{i}=\frac{1}{2}\log\frac{\sigma_{D}^{2}}{\sigma_{T}^{2}}-\frac{1}{2}\left[\frac{{\left(x_{i}-s_{T}\right)}^{2}}{\sigma_{T}^{2}}-\frac{{\left(x_{i}-s_{D}\right)}^{2}}{\sigma_{D}^{2}}\right]$$ (5)

#### Learning

We modeled perceptual learning as a decrease in orientation uncertainty over time, according to an exponential function,

$$\sigma =\sigma_{0}(1+e^{-\tau t})$$ (6)

where σ is the uncertainty, σ_0_ is the initial (pre-training) uncertainty, τ is the learning rate, and t is time in sessions. Exponential functions are commonly used to describe the time course of perceptual learning (e.g., [44,66,67]).

#### Model d′

To calculate d′ for the search model, we simulated an experiment with 4,000 trials (50% target present) by randomly generating orientation measurements (x_i_) for each stimulus in the display for each trial. The orientation measurements were drawn from normal distributions with means and standard deviations that depended on whether the stimulus was near-cardinal or oblique (mapped to target or distractor depending on the group, Eq 4).

From the simulated orientation measurements and search model values for σ_T_ and σ_D_, we calculated the decision variable d on each trial according to Eqs 2 and 5. If d>0, the model response was classified as target present; otherwise it was classified as target absent. The simulated responses were used to calculate d′ according to the standard formula (the same as for the behavioral data).

#### Model parameters

We assumed that the two groups started with the same levels of uncertainty about the stimulus orientations. The uncertainty could be different for near-cardinal and oblique orientations but did not depend on which one was assigned to be the target, modeling the preexisting reliability of each orientation.

We fit four different versions of the model:

1 Reliability (R) model. Initial reliability can differ for near-cardinal and oblique orientations, but learning is the same for all orientations in the two groups. This model has three parameters: the initial uncertainties of near-cardinal and oblique orientations, σ_cardinal_ and σ_oblique_, and the single learning rate, τ_σ_. **Table 1** shows the orientation uncertainties used for the different groups.

**Table 1:** Mapping for target and distractor orientation uncertainties.

	σ_T_	σ_D_
Near-cardinal group	σ_cardinal_	σ_oblique_
Oblique group	σ_oblique_	σ_cardinal_

2 Learning (L) model. Initial reliability is fixed to the same value for cardinal and oblique orientations, but the learning rate can differ for target and distractor stimuli. Note that learning rates depend only on the role of the stimulus in the task (target vs. distractor [43]) and not on orientation per se. This models the idea that the learning rate depends only on the amount of exposure to a given stimulus orientation. The model has three parameters: the initial uncertainty σ, and the learning rates for the target and distractor, τ_σT_ and τ_σD_.
3 Reliability-and-Learning (RL) model. Initial reliability can differ for near-cardinal and oblique orientations, and learning rates can differ for targets and distractors. This model has four parameters: the initial uncertainties of near-cardinal and oblique orientations, σ_cardinal_ and σ_oblique_, and the learning rates for each sigma: τ_σT_ and τ_σD_.
4 Reliability-Learning-Group (RLG) model. Initial reliability can differ for near-cardinal and oblique orientations, and learning rates are independent for the two groups of observers. Thus, learning rates depend on whether the near-cardinal or oblique orientation is the target or distractor. This model has six parameters: the initial uncertainties of near-cardinal and oblique orientations, σ_cardinal_ and σ_oblique_, and the learning rates for each sigma in each group: τ_σTcardinal_, τ_σDcardinal_, τ_σToblique_, and τ_σDoblique_.

#### Model fitting

We simultaneously fit the behavioral data from the two groups of observers (near-cardinal group and oblique group) from seven experiment sessions: the six training sessions and the orientation transfer test. We took the behavioral d′ for each session, averaged across SOAs and across observers, as our measure of visual search performance, resulting in 14 data points. We averaged across SOAs, because the transfer asymmetries we were interested in explaining were independent of SOA and because the model did not have a dynamic component that would be appropriate to capture SOA effects. To fit the data, we minimized the sum of squared errors between the model d′ and the behavioral d′ using the Matlab function fminsearch. On each iteration of the optimization, a full experiment was stochastically simulated to calculate the model d′, as described above. Several starting points for the parameters were used to check the consistency of the model fits (**S2 Fig**).

#### Model evaluation and noise ceiling

We used AICc for model comparison and cross-validated r^2^ to measure a model’s goodness of fit, free from overfitting [68]. To calculate cross-validated r^2^, we used a leave-one-out procedure. On each iteration, one observer was held out and the model was fit to the two group means based on the remaining 9 observers. This fit served as the prediction for the held out observer. We then computed r^2^ for each observer from a Pearson’s r correlation between the predicted and true data from that observer, and took the group mean as our measure of cross-validated r^2^.

The maximum achievable cross-validated r^2^ is limited by measurement noise, individual variability, and other sources of noise in the data. Therefore, to provide a benchmark for our r^2^ values, we calculated a noise ceiling (a lower and upper bound for the maximum achievable cross-validated r^2^) according to the procedure described by [69]. The upper bound is defined as the mean r^2^ for the correlation of each observer's data with the group mean. The lower bound is defined as the mean r^2^ for the correlation of each observer's data with the group mean when that observer is left out. We computed separate means for each group and correlated each observer’s data with the appropriate group mean; so group means were based on 5 observers for the upper bound and 4 observers for the lower bound.

## Supporting information

S1 Table Wilcoxon rank sum tests for transfer and specificity. (PDF)

S1 Fig Model performance for the near-cardinal (left column) and oblique (right column) groups. Curves show the Reliability (R), Learning (L) and Reliability-Learning-Group (RLG) model fits to the sensitivity data (black points) averaged across SOAs for each training day. Error bars are standard error of the mean.
(PDF)

S2 Fig Reliability-and-Learning model fitting. Scatter plots for each pair of the Reliability-and-Learning model parameter values for each optimization starting point. Dot color corresponds to the R^2^ of the fit. The red star represents the best fit, which is reported in the main text. The starting points for σ_c_ and σ_o_ were uniformly sampled from 1 to 20. Values of σ above 20 always resulted in zero hit-rate in the first session so could not be used. The starting points for τ_σT_ and τ_σD_ were uniformly sampled from 0 to 2. Bars show marginal parameter distributions for each R^2^ level, normalized to sum to 1. c = near-cardinal, o = oblique, T = target, D = distractor.
(PDF)

S1 Text The derivation of the optimal decision rule *d*_*i*_ for a single display element. (PDF)

## References

[1] Green CS, Bavelier D. Learning, attentional control, and action video games. Current Biology. 2012;22: R197–R206. doi: 10.1016/j.cub.2012.02.012 22440805 10.1016/j.cub.2012.02.012 PMC3461277

[2] Li J, Thompson B, Deng D, Chan LYL, Yu M, Hess RF. Dichoptic training enables the adult amblyopic brain to learn. Current Biology. 2013;23: R308–R309. doi: 10.1016/j.cub.2013.01.059 23618662 10.1016/j.cub.2013.01.059

[3] Shibata K, Kawato M, Watanabe T, Sasaki Y. Monocular deprivation boosts long-term visual plasticity. Current Biology. 2012;22: R291–R292. doi: 10.1016/j.cub.2012.03.010 22575461 10.1016/j.cub.2012.03.010 PMC3536019

[4] Das A, Tadin D, Huxlin KR. Beyond blindsight: Properties of visual relearning in cortically blind fields. Journal of Neuroscience. 2014;34: 11652–11664. doi: 10.1523/JNEUROSCI.1076-14.2014 25164661 10.1523/JNEUROSCI.1076-14.2014 PMC4145170

[5] Hussain Z, Webb BS, Astle AT, McGraw PV. Perceptual learning reduces crowding in amblyopia and in the normal periphery. Journal of Neuroscience. 2012;32: 474–480. doi: 10.1523/JNEUROSCI.3845-11.2012 22238083 10.1523/JNEUROSCI.3845-11.2012 PMC3428833

[6] Levi DM, Polat U. Neural plasticity in adults with amblyopia. Proceedings of the National Academy of Sciences. 1996;93: 6830–6834. 10.1073/pnas.93.13.6830 PMC39113 8692904

[7] Polat U, Ma-Naim T, Belkin M, Sagi D. Improving vision in adult amblyopia by perceptual learning. Proceedings of the National Academy of Sciences. 2004;101: 6692–6697. doi: 10.1073/pnas.0401200101 15096608 10.1073/pnas.0401200101 PMC404107

[8] Polat U, Schor C, Tong J-L, Zomet A, Lev M, Yehezkel O, et al Training the brain to overcome the effect of aging on the human eye. Scientific Reports. 2012;2: 278 doi: 10.1038/srep00278 22363834 10.1038/srep00278 PMC3284862

[9] Sagi D. Perceptual learning in vision research. Vision Research. 2011;51: 1552–1566. doi: 10.1016/j.visres.2010.10.019 20974167 10.1016/j.visres.2010.10.019

[10] Ahissar M, Hochstein S. Task-difficulty and the specificity of perceptual-learning. Nature. 1997;387: 401–406. doi: 10.1038/387401a0 9163425 10.1038/387401a0

[11] Fahle M. Perceptual learning: A case for early selection. Journal of Vision.2004;4(10): 4 doi: 10.1167/4.10.4 15595892 10.1167/4.10.4

[12] Coppola DM, White LE, Fitzpatrick D, Purves D. Unequal representation of cardinal and oblique contours in ferret visual cortex. Proceedings of the National Academy of Sciences. 1998;95: 2621–2623. 10.1073/pnas.95.5.2621 PMC19437 9482936

[13] Li B, Peterson MR. Oblique effect: A neural basis in the visual cortex. Journal of Neurophysiology. 2003;90: 204–217. doi: 10.1152/jn.00954.2002 12611956 10.1152/jn.00954.2002

[14] van Bergen RS, Ma WJ, Pratte MS, Jehee JFM. Sensory uncertainty decoded from visual cortex predicts behavior. Nature Neuroscience; 2015;18: 1728–1730. doi: 10.1038/nn.4150 26502262 10.1038/nn.4150 PMC4670781

[15] Furmanski CS, Engel SA. An oblique effect in human primary visual cortex. Nature Neuroscience; 2000;3: 535–536. doi: 10.1038/75702 10816307 10.1038/75702

[16] Girshick AR, Landy MS, Simoncelli EP. Cardinal rules: visual orientation perception reflects knowledge of environmental statistics. Nature Neuroscience; 2011;14: 2831–932. doi: 10.1038/nn.2831 21642976 10.1038/nn.2831 PMC3125404

[17] Appelle S. Perception and discrimination as a function of stimulus orientation: The “oblique effect” in man and animals. Psychological Bulletin. 1972;78: 266–278. 4562947 10.1037/h0033117

[18] Carrasco M, Frieder KS. Cortical magnification neutralizes the eccentricity effect in visual search. Vision Research; 1997;37: 63–82. doi: 10.1016/S0042-6989(96)00102-2 9068831 10.1016/s0042-6989(96)00102-2

[19] Carrasco M, McLean TL, Katz SM, Frieder KS. Feature asymmetries in visual search: Effects of display duration, target eccentricity, orientation and spatial frequency. Vision Research. 1998;38: 347–374. doi: 10.1016/S0042-6989(97)00152-1 9536360 10.1016/s0042-6989(97)00152-1

[20] Vincent BT. Search asymmetries: Parallel processing of uncertain sensory information. Vision Research. 2011;51: 1741–1750. doi: 10.1016/j.visres.2011.05.017 21664919 10.1016/j.visres.2011.05.017

[21] Foster DH, Ward PA. Asymmetries in oriented-line detection indicate two orthogonal filters in early vision. Proceedings of the Royal Society of London B: Biological Sciences; 1991;243: 75–81. doi: 10.1098/rspb.1991.0013 1673245 10.1098/rspb.1991.0013

[22] Treisman A, Souther J. Search asymmetry: A diagnostic for preattentive processing of separable features. Journal of Experimental Psychology: General. 1985;114: 285–310. doi: 10.1037/0096-3445.114.3.285 3161978 10.1037//0096-3445.114.3.285

[23] De Valois RL, Albrecht DG, Thorell LG. Spatial frequency selectivity of cells in macaque visual cortex. Vision Research. 1982;22: 545–559. doi: 10.1016/0042-6989(82)90113-4 7112954 10.1016/0042-6989(82)90113-4

[24] Ahissar M, Hochstein S. The reverse hierarchy theory of visual perceptual learning. Trends in Cognitive Sciences. 2004;8: 457–464. doi: 10.1016/j.tics.2004.08.011 15450510 10.1016/j.tics.2004.08.011

[25] Jeter PE, Dosher BA, Lu Z-L. Task precision at transfer determines specificity of perceptual learning. Journal of Vision. 2009;9(3): 1 doi: 10.1167/9.3.1 19757940 10.1167/9.3.1 PMC4964592

[26] Ma WJ, Navalpakkam V, Beck JM, Berg RVD, Pouget A. Behavior and neural basis of near-optimal visual search. Nature Neuroscience. 2011;14: 783–790. doi: 10.1038/nn.2814 21552276 10.1038/nn.2814 PMC3713779

[27] Morey RD. Confidence intervals from normalized data: A correction to Cousineau (2005). Tutorials in Quantitative Methods for Psychology. 2008;4: 61–64. doi: 10.3758/s13414-012-0291-2

[28] Mazyar H, van den Berg R, Ma WJ. Does precision decrease with set size? Journal of Vision. 2012;12(6): 10 doi: 10.1167/12.6.10 22685337 10.1167/12.6.10 PMC3677801

[29] Dosher BA, Jeter P, Liu J, Lu Z-L. An integrated reweighting theory of perceptual learning. Proceedings of the National Academy of Sciences. 2013;110: 13678–13683. doi: 10.1073/pnas.1312552110 23898204 10.1073/pnas.1312552110 PMC3746919

[30] Zhang J-Y, Zhang G-L, Xiao L-Q, Klein SA, Levi DM, Yu C. Rule-based learning explains visual perceptual learning and its specificity and transfer. Journal of Neuroscience. 2010;30: 12323–12328. doi: 10.1523/JNEUROSCI.0704-10.2010 20844128 10.1523/JNEUROSCI.0704-10.2010 PMC3842491

[31] Hung S-C, Seitz AR. Prolonged training at threshold promotes robust retinotopic specificity in perceptual learning. Journal of Neuroscience. 2014;34: 8423–8431. doi: 10.1523/JNEUROSCI.0745-14.2014 24948798 10.1523/JNEUROSCI.0745-14.2014 PMC4061387

[32] Donovan I, Szpiro S, Carrasco M. Exogenous attention facilitates location transfer of perceptual learning. Journal of Vision. 2015;15(10): 11 doi: 10.1167/15.10.11 26426818 10.1167/15.10.11 PMC4594468

[33] Szpiro SFA, Carrasco M. Exogenous attention enables perceptual learning. Psychological Science. 2015;26: 1854–1862. doi: 10.1177/0956797615598976 26502745 10.1177/0956797615598976 PMC4695399

[34] Yashar A, Chen J, Carrasco M. Rapid and long-lasting reduction of crowding through training. Journal of Vision. 2015;15(10): 15 doi: 10.1167/15.10.15 26583278 10.1167/15.10.15 PMC4669205

[35] Ahissar M, Laiwand R, Kozminsky G, Hochstein S. Learning pop-out detection: Building representations for conflicting target-distractor relationships. Vision Research. 1998;38: 3095–3107. 9893818 10.1016/s0042-6989(97)00449-5

[36] Yashar A, Carrasco M. Rapid and long-lasting learning of feature binding. Cognition. 2016;154: 130–138. doi: 10.1016/j.cognition.2016.05.019 27289484 10.1016/j.cognition.2016.05.019 PMC4939117

[37] Karni A, Sagi D. Where practice makes perfect in texture discrimination: Evidence for primary visual cortex plasticity. Proceedings of the National Academy of Sciences. 1991;88: 4966–4970. 10.1073/pnas.88.11.4966 PMC51788 2052578

[38] Harris H, Gliksberg M, Sagi D. Generalized perceptual learning in the absence of sensory adaptation. Current Biology. 2012;22: 1813–1817. doi: 10.1016/j.cub.2012.07.059 22921366 10.1016/j.cub.2012.07.059

[39] Schwartz S, Maquet P, Frith C. Neural correlates of perceptual learning: A functional MRI study of visual texture discrimination. Proceedings of the National Academy of Sciences. 2002;99: 17137–17142. doi: 10.1073/pnas.242414599 12446842 10.1073/pnas.242414599 PMC139282

[40] Walker MP, Stickgold R, Jolesz FA, Yoo S-S. The functional anatomy of sleep-dependent visual skill learning. Cerebral Cortex. 2005;15: 1666–1675. doi: 10.1093/cercor/bhi043 15703253 10.1093/cercor/bhi043

[41] Yotsumoto Y, Watanabe T, Sasaki Y. Different dynamics of performance and brain activation in the time course of perceptual learning. Neuron. 2008;57: 827–833. doi: 10.1016/j.neuron.2008.02.034 18367084 10.1016/j.neuron.2008.02.034 PMC2735208

[42] Shibata K, Sagi D, Watanabe T. Two-stage model in perceptual learning: toward a unified theory. Annals of the New York Academy of Sciences. 2014;1316: 18–28. doi: 10.1111/nyas.12419 24758723 10.1111/nyas.12419 PMC4103699

[43] Le Dantec CC, Melton EE, Seitz AR. A triple dissociation between learning of target, distractors, and spatial contexts. Journal of Vision. 2012;12(2): 5 doi: 10.1167/12.2.5 22306889 10.1167/12.2.5

[44] Ahissar M, Hochstein S. Learning pop-out detection: specificities to stimulus characteristics. Vision Research. 1996;36: 3487–3500. 8977015 10.1016/0042-6989(96)00036-3

[45] Rubenstein BS, Sagi D. Spatial variability as a limiting factor in texture-discrimination tasks: implications for performance asymmetries. Journal of the Optical Society of America A, 1990;7: 1632–1643. doi: 10.1364/JOSAA.7.001632 10.1364/josaa.7.001632 2213287

[46] Treisman A, Gormican S. Feature analysis in early vision: Evidence from search asymmetries. Psychological Review. 1988;95: 15–48. 3353475 10.1037/0033-295x.95.1.15

[47] Treisman A. Preattentive processing in vision. Computer Vision, Graphics, and Image Processing. 1985;31: 156–177. doi: 10.1016/S0734-189X(85)80004-9

[48] Li Z. Contextual influences in V1 as a basis for pop out and asymmetry in visual search. Proceedings of the National Academy of Sciences. 1999;96: 10530–10535. doi: 10.1073/pnas.96.18.10530 10.1073/pnas.96.18.10530 PMC17923 10468643

[49] Wolfe JM. Asymmetries in visual search: An introduction. Perception & Psychophysics. 2001;63: 381–389. doi: 10.3758/BF03194406 11414127 10.3758/bf03194406

[50] Adab HZ, Vogels R. Practicing coarse orientation discrimination improves orientation signals in macaque cortical area V4. Current Biology. 2011;21: 1661–1666. doi: 10.1016/j.cub.2011.08.037 21962714 10.1016/j.cub.2011.08.037

[51] Raiguel S, Vogels R, Mysore SG, Orban GA. Learning to see the difference specifically alters the most informative V4 neurons. Journal of Neuroscience. 2006;26: 6589–6602. doi: 10.1523/JNEUROSCI.0457-06.2006 16775147 10.1523/JNEUROSCI.0457-06.2006 PMC6674023

[52] Schoups A, Vogels R, Qian N, Orban G. Practising orientation identification improves orientation coding in V1 neurons. Nature. 2001;412: 549–553. doi: 10.1038/35087601 11484056 10.1038/35087601

[53] Yang T, Maunsell JHR. The effect of perceptual learning on neuronal responses in monkey visual area V4. Journal of Neuroscience. 2004;24: 1617–1626. doi: 10.1523/JNEUROSCI.4442-03.2004 14973244 10.1523/JNEUROSCI.4442-03.2004 PMC6730469

[54] Carrasco M, P Talgar C, Cameron EL. Characterizing visual performance fields: Effects of transient covert attention, spatial frequency, eccentricity, task and set size. Spatial Vision. 2001;15: 61–75. doi: 10.1163/15685680152692015 11893125 10.1163/15685680152692015 PMC4332623

[55] Greenwood JA, Szinte M, Sayim B, Cavanagh P. Variations in crowding, saccadic precision, and spatial localization reveal the shared topology of spatial vision. Proceedings of the National Academy of Sciences. 2017;114: E3573–E3582. doi: 10.1073/pnas.1615504114 28396415 10.1073/pnas.1615504114 PMC5410794

[56] Deveau J, Lovcik G, Seitz AR. The therapeutic benefits of perceptual learning. Current Trends in Neurology. 2013;7: 39 25580062 PMC4286158

[57] Franceschini S, Gori S, Ruffino M, Viola S, Molteni M, Facoetti A. Action video games make dyslexic children read better. Current Biology. 2013;23: 462–466. doi: 10.1016/j.cub.2013.01.044 23453956 10.1016/j.cub.2013.01.044

[58] Sterkin A, Yehezkel O, Lev M, Doron R, Fried M, Levy Y, et al Perceptual learning improves near vision in pilots with eye aging. Journal of Vision. 2014;14: 1173–1173.

[59] Greenwood JA, Edwards M. An oblique effect for transparent-motion detection caused by variation in global-motion direction-tuning bandwidths. Vision Research. 2007;47: 1411–1423. doi: 10.1016/j.visres.2007.02.004 17391725 10.1016/j.visres.2007.02.004

[60] Virsu V, Rovamo J. Visual resolution, contrast sensitivity, and the cortical magnification factor. Experimental Brain Research. 1979;37: 475–494. doi: 10.1007/BF00236818 520438 10.1007/BF00236818

[61] Schneider W, Eschman A, Zuccolotto A. E-Prime: User's guide Psychology Software Incorporated; 2002.

[62] Ahissar M, Hochstein S. Attentional control of early perceptual learning. Proceedings of the National Academy of Sciences. 1993;90: 5718–5722. doi: 10.1073/pnas.90.12.5718 10.1073/pnas.90.12.5718 PMC46793 8516322

[63] Song Y, Sun L, Wang Y, Zhang X, Kang J, Ma X, et al The effect of short-term training on cardinal and oblique orientation discrimination: An ERP study. International Journal of Psychophysiology. 2010;75: 241–248. doi: 10.1016/j.ijpsycho.2009.11.007 19995581 10.1016/j.ijpsycho.2009.11.007

[64] Vogels R, Orban GA. The effect of practice on the oblique effect in line orientation judgments. Vision Research. 1985;25: 1679–1687. doi: 10.1016/0042-6989(85)90140-3 3832592 10.1016/0042-6989(85)90140-3

[65] Westheimer G, Lavian J. Perceptual learning of orientation judgments in oblique meridians. Attention, Perception, & Psychophysics. 2013;75: 1252–1259. doi: 10.3758/s13414-013-0478-1 23709066 10.3758/s13414-013-0478-1

[66] Green CS, Kattner F, Siegel MH, Kersten D, Schrater PR. Differences in perceptual learning transfer as a function of training task. Journal of Vision. 2015;15: 5–5. doi: 10.1167/15.10.5 26305737 10.1167/15.10.5

[67] Leibowitz N, Baum B, Enden G, Karniel A. The exponential learning equation as a function of successful trials results in sigmoid performance. Journal of Mathematical Psychology. 2010;54: 338–340. doi: 10.1016/j.jmp.2010.01.006

[68] Kay KN, Winawer J, Mezer A, Wandell BA. Compressive spatial summation in human visual cortex. Journal of Neurophysiology. 2013;110: 481–494. doi: 10.1152/jn.00105.2013 23615546 10.1152/jn.00105.2013 PMC3727075

[69] Wardle SG, Kriegeskorte N, Grootswagers T, Khaligh-Razavi S-M, Carlson TA. Perceptual similarity of visual patterns predicts dynamic neural activation patterns measured with MEG. NeuroImage. 2016;132: 59–70. doi: 10.1016/j.neuroimage.2016.02.019 26899210 10.1016/j.neuroimage.2016.02.019

