# Supplementary material for: Feature reliability determines specificity and transfer of perceptual learning in orientation search
Source: PLoS Comput Biol. 2017 Dec 14;13(12):e1005882. doi: 10.1371/journal.pcbi.1005882 (PMC5746251; doi:10.1371/journal.pcbi.1005882)
Supplement: S1 Table — (PDF) [file pcbi.1005882.s001.pdf]

S1 Table. Wilcoxon rank sum tests for transfer and specificity

Test	Comparison	p-value
Oblique group transfer	Near-cardinal group day 1 vs. oblique group transfer test	$p = 0.004$
Near-cardinal group transfer	Oblique group day 1 vs. near-cardinal group transfer test	$p = 0.048$
Oblique group partial specificity	Near-cardinal group day 6 vs. oblique group transfer test	$p = 0.032$
Near-cardinal group partial specificity	Oblique group day 6 vs. near-cardinal group transfer test	$p > 0.4$

Table. Pairwise comparisons were confirmed using Wilcoxon rank sum tests. These correspond to the t-tests in the transfer and specificity section of the Results.
