# Supplementary material for: Feature reliability determines specificity and transfer of perceptual learning in orientation search
Source: PLoS Comput Biol. 2017 Dec 14;13(12):e1005882. doi: 10.1371/journal.pcbi.1005882 (PMC5746251; doi:10.1371/journal.pcbi.1005882)
Supplement: S1 Fig — Curves show the Reliability (R), Learning (L) and Reliability-Learning-Group (RLG) model fits to the sensitivity data (black points) averaged across SOAs for each training day. Error bars are standard error of the mean. (PDF) [file pcbi.1005882.s002.pdf]

S1 Fig. Model performance for the near-cardinal (left column) and oblique (right column) groups.

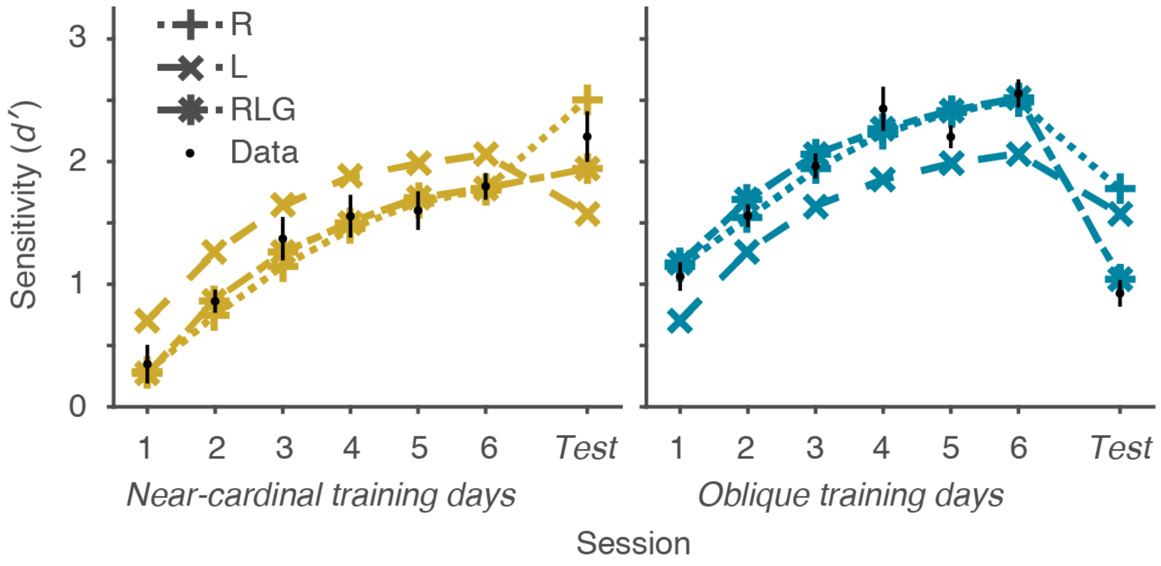

Fig. Model performance for the near-cardinal (left column) and oblique (right column) groups. Curves show the Reliability (R), Learning (L) and Reliability-Learning-Group (RLG) model fits to the sensitivity data (black points) averaged across SOAs for each training day. Error bars are standard error of the mean.
