# Supplementary material for: Feature reliability determines specificity and transfer of perceptual learning in orientation search
Source: PLoS Comput Biol. 2017 Dec 14;13(12):e1005882. doi: 10.1371/journal.pcbi.1005882 (PMC5746251; doi:10.1371/journal.pcbi.1005882)
Supplement: S2 Fig — Scatter plots for each pair of the Reliability-and-Learning model parameter values for each optimization starting point. Dot color corresponds to the R2 of the fit. The red star represents the best fit, which is reported in the main text. The starting points for σc and σo were uniformly sampled from 1 to 20. Values of σ above 20 always resulted in zero hit-rate in the first session so could not be used. The starting points for τσT and τσD were uniformly sampled from 0 to 2. Bars show marginal parameter distributions for each R2 level, normalized to sum to 1. c = near-cardinal, o = oblique, T = target, D = distractor. (PDF) [file pcbi.1005882.s003.pdf]

S2 Fig. Reliability-and-Learning model fitting

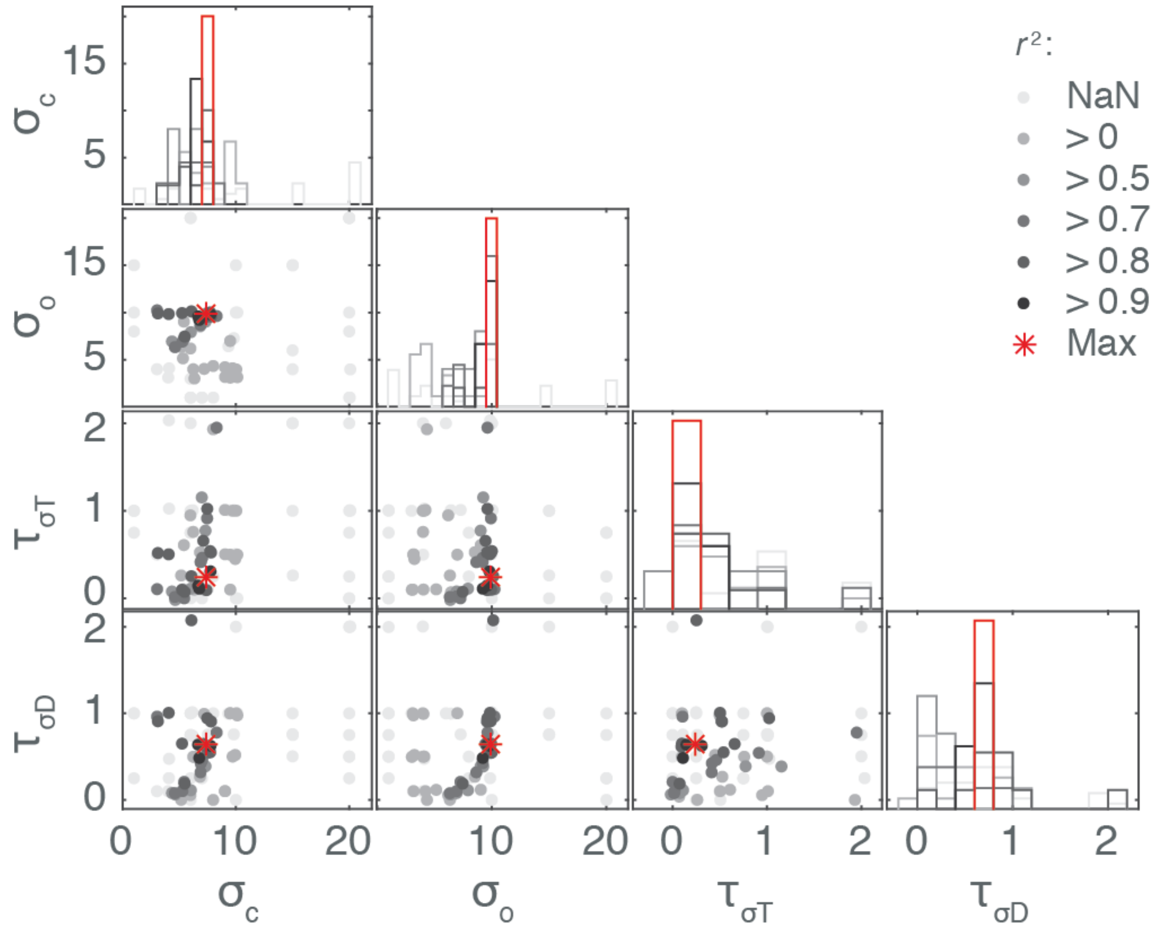

Fig. Reliability-and-Learning model fitting. Scatter plots for each pair of the Reliability-and-Learning model parameter values for each optimization starting point. Dot color corresponds to the R^2 of the fit. The red star represents the best fit, which is reported in the main text. The starting points for σ_c and σ_o were uniformly sampled from 1 to 20. Values of σ above 20 always resulted in zero hit-rate in the first session so could not be used. The starting points for $\tau_{\sigma T}$ and $\tau_{\sigma D}$ were uniformly sampled from 0 to 2. Bars show marginal parameter distributions for each R^2 level, normalized to sum to 1. c = near-cardinal, o = oblique, T = target, D = distractor.
