# Supplementary material for: Feature reliability determines specificity and transfer of perceptual learning in orientation search
Source: PLoS Comput Biol. 2017 Dec 14;13(12):e1005882. doi: 10.1371/journal.pcbi.1005882 (PMC5746251; doi:10.1371/journal.pcbi.1005882)
Supplement: S1 Text — (PDF) [file pcbi.1005882.s004.pdf]

S1 Text. The derivation of the optimal decision rule d_i for a single display element

The derivation of the optimal decision rule d_i for a single display element (Equation 5 in the main text) is as follows:

$$d_i = \log \frac{p(x \mid \text{target present})}{p(x \mid \text{target absent})}$$

$$= \log\left(\frac{1}{\sqrt{(2\pi\sigma_T^2)}} e^{-\frac{(x-s_T)^2}{2\sigma_T^2}} \right) - \log\left(\frac{1}{\sqrt{(2\pi\sigma_D^2)}} e^{-\frac{(x-s_D)^2}{2\sigma_D^2}} \right)$$

$$= \frac{1}{2}\log\frac{\sigma_D^2}{\sigma_T^2} - \frac{(x-s_T)^2}{2\sigma_T^2} + \frac{(x-s_D)^2}{2\sigma_D^2}$$

$$= \frac{1}{2}\log\frac{\sigma_D^2}{\sigma_T^2} - \frac{1}{2}\left(\frac{(x-s_T)^2}{\sigma_T^2} - \frac{(x-s_D)^2}{\sigma_D^2} \right)$$
